# Tranilast Directly Targets NLRP3 to Protect Melanocytes From Keratinocyte-Derived IL-1β Under Oxidative Stress

**DOI:** 10.3389/fcell.2020.00588

**Published:** 2020-07-10

**Authors:** Tongtian Zhuang, Shuli Li, Xiuli Yi, Sen Guo, Yinghan Wang, Jianru Chen, Ling Liu, Zhe Jian, Tianwen Gao, Pan Kang, Chunying Li

**Affiliations:** Department of Dermatology, Xijing Hospital, Fourth Military Medical University, Xi’an, China

**Keywords:** vitiligo, tranilast, NLRP3, IL-1β, keratinocyte, melanocyte

## Abstract

The activation of NLRP3 inflammasome-IL-1β pathway in keratinocytes contributes to the melanocyte death via autoimmunity-dependent manner in vitiligo. As a safe small-compound drug employed frequently in clinic, tranilast (TR) is newly reported to block the activation of NLRP3 inflammasome in macrophage. Nevertheless, whether keratinocyte-derived IL-1β damages melanocytes in an autoimmunity-independent way and whether TR could ameliorate the melanocyte damage via inhibiting the NLRP3-IL-1β pathway in keratinocyte still are not clear. In the present study, we initially found that TR could impede the secretion of IL-1β from keratinocytes by interfering the NLRP3 oligomerization. More importantly, we illustrated that TR could decrease the melanocyte apoptosis, improve the melanogenesis, and have the capacity to optimize the melanosome translocation by abolishing the keratinocyte-derived IL-1β. Additionally, TR could mitigate the secretion of inflammatory cytokines such as IL-6, IL-8, TNF-α, and IL-18 in keratinocytes under oxidative stress. In short, our data indicate that IL-1β plays detrimental roles in the melanocyte survival, melanogenesis, melanosome translocation and the secretion of inflammatory cytokines, and TR could be a promising therapeutic strategy in vitiligo by attenuating the keratinocyte-derived IL-1β under oxidative stress.

## Introduction

Vitiligo is an acquired disfiguring inflammatory disease characterized by the destruction of melanocytes (Picardo et al., 2015), which brings colossal psychological plight to patients (Ongenae et al., 2006) and is still lack of the effective therapeutic strategy (Frisoli and Harris, 2017). It has been confirmed that oxidative stress plays a critical role in the pathogenesis of vitiligo, which is supported by the accumulation of hydrogen peroxide (H_2_O_2_) in epidermis of vitiligo patients (Schallreuter et al., 1999). A large number of studies have shown that oxidative stress can induce the apoptosis of melanocytes and the dysfunction of melanogenesis (Mosenson et al., 2012). Importantly, recent researches have demonstrated that keratinocytes contribute to the melanocyte destruction in vitiligo pathogenesis (Shi et al., 2016).

Previous studies found that overexpression of miR-25 in stressed keratinocytes interferes with the production and secretion of stem cell factor and basic fibroblast growth factor from keratinocytes, thus facilitating melanocyte loss in patients with vitiligo (Shi et al., 2016). Besides, the keratinocyte-derived chemokines, especially the chemokine (C-X-C motif) ligand (CXCL)10 and CXCL16, have been proved to aggravate the melanocytes loss via attracting autoreactive melanocyte-specific CD8^+^ T cells (Rashighi et al., 2014; Li et al., 2017). These all back that oxidative stress not only exerts its deleterious impacts directly on melanocytes in ways such as disrupting the cell homeostasis and promoting apoptosis, but also indirectly impairs melanocytes via mediating keratinocyte which is the predominant component in epidermis.

Recently, we confirmed that oxidative stress promoted proinflammatory cytokine interleukin-1β (IL-1β) secretion from keratinocytes via nucleotide-binding oligomerization domain, leucine-rich repeat and pyrin domain-containing protein 3 (NLRP3) – caspase1 signaling pathway (Li et al., 2019). NLRP3 is a member of nucleotide binding domain-like receptor (NLR) family, together with apoptosis-associated speck-like protein containing a CARD (ASC) and caspase1, forming NLRP3 inflammasome complex, which responds to microbes and danger signals and activates proinflammatory cytokines including IL-1β (Jo et al., 2016; Chung et al., 2019). Increasing evidences indicated that the dysregulation of NLRP3 inflammasome plays a critical role in the pathogenesis of several inflammatory diseases, such as atypical dermatitis (Darakhshan and Pour, 2015), inflammatory bowel disease (Cocco et al., 2017) and neurodegenerative diseases (Heneka et al., 2013). Jinjin Dong et al. also reported that the interleukin-22 (IL-22) induced the production of IL-1β via the NLRP3-caspase1 signaling pathway in keratinocytes, and what’s striking is that the conditional medium derived from IL-22-stimulated keratinocytes facilitated the apoptosis of melanocytes and hindered the melanogenesis and melanosome translocation (Dong et al., 2017). These studies indicated that the keratinocytes-derived IL-1β may contribute to the melanocyte destruction under oxidative stress and NLRP3 inflammasome can be regarded as a potential drug target for the treatment of vitiligo.

Tranilast (N- [3′,4′-dimethoxycinnamoyl]-anthranilic acid, TR) is a common anti-allergic drug clinically used to inhibit the IgE-induced histamine release from mast cells (Huang et al., 2018). A recent study has suggested that TR suppressed the assembly of NLRP3 inflammasome by blocking NLRP3 oligomerization, which further ablated the secretion of IL-1β in macrophages (Huang et al., 2018). Given this effect of TR, it has been clinically used in a variety of inflammatory diseases, including atypical dermatitis and hypertrophic scars (Darakhshan and Pour, 2015). Hence, we hypothesized that under the oxidative stress, TR could impede the production of IL-1β in keratinocytes and further abolish the detrimental effects of IL-1β on melanocytes in vitiligo.

In the present study, we initially found that TR inhibited the activation of NLRP3 inflammasome by intervening its oligomerization in keratinocytes under the oxidative stress. Sequentially, we verified that TR reversed the melanocyte apoptosis and had the capacity to improve the melanogenesis and melanosome translocation via mitigating IL-1β secretion from keratinocytes. Additionally, we manifested that TR pretreatment could significantly palliate the severity of inflammation in epidermis under oxidative stress. These results supported TR as an effective therapeutic treatment for vitiligo.

## Materials and Methods

### Cell Culture

Normal Human keratinocytes (NHKs) were isolated from skin samples collected from subjects during plastic surgery in Xijing hospital and cultured in keratinocyte-SFM (Gibco, United States). Cells were grown to 80% confluency and used in 3–5 passages. Normal human keratinocyte cell line HaCaT (purchased from ATCC and authenticated by STR profiling recently) was cultured in Modified Medium RPIM 1640 with 10% FBS and L-glutamine (Invitrogen, Carlsbad, CA, United States) without mycoplasma contamination. The immortalized normal human epidermal melanocyte cell line PIG1 (a gift from Dr. Caroline Le Poole, Loyola University Chicago, Maywood, IL, United States and authenticated by STR profiling recently) was cultured as previously described without mycoplasma contamination. Cells were cultured in a humidified atmosphere with 5% CO_2_ at 37°C.

### CCK8 Assay

The IC50 of TR on HaCaT and cell viability were determined by CCK8 kit (Lot. C008, 7Sea biotech, China) according to the manufacturer’s instructions. HaCaT cells were seeded into 96-well plates at the density of 5000 each well, and then treated with TR (Lot. S1439, Selleck) for 24 h with different concentrations. Next, cells were incubated with 100 (μL fresh medium with 10 (μL CCK8 solution for 2 h at 37°C and optical density (OD) was measured at 450 nm by Model 680 Microplate Reader (Bio-Rad, United States).

### ELISA

Human IL-1β Quantitative ELISA kit (Lot. DLB50, R&D Systems, United States) were used to analyze cell culture supernatant samples according to the manufacturer’s instructions. Briefly, cell culture supernatant samples were equilibrated to the room temperature from stored deep-frozen (−80°C) prior to use. Next, all samples and the diluted standard antibody samples were added into appropriate wells. After incubating for 2 h at room temperature, aspirating the content of every wells and washing well with 400 μL 1 × Washing solution for three times. Then 200 μL human IL-1β Conjugate was added into each well for 1 h at room temperature. After washing for 3 times, adding 200 μL Substrate Solution to all wells for 20 min at room temperature, protecting from light. Last, adding 50 μL of Stop Solution to each well. Determine the optical density of each well within 30 min at 450 nm.

### Western-Blot Assay

After washed and lysed, the total protein was collected and the concentration was measured with BCA Protein Assay kit (Pierce, Rockford, IL, United States). Equal amounts of protein were separated by 10% SDS-PAGE (Bio-Rad, United States) and transferred to Polyvinylidene difluoride membranes (Millipore, Billerica, United States). Page Ruler Plus Prestained Protein Ladder (Fermentas, Hanover, United States) was used to confirm protein electrophoresis and transfer. Then the phosphorylation form of molecule was blocked with 5% bovine serum albumin, while the total form of molecule was blocked with 5% non-fat dry milk for 2 h. After washed with TBST (TBS + 0.1% [v/v] Tween-20) transitorily, the membranes were incubated with primary antibodies against β-actin (8H10D10) (1:5000, Lot. 3700, Cell Signaling Technology), NLRP3 (1:1000, Lot. ab214185, Abcam) Pro-caspase1 (D7F10) (1:1000, Lot. 3866, Cell Signaling Technology), Pro-IL-1β (3A6) (1:1000, Lot. 12703, Cell Signaling Technology), Cleaved-caspase1 (p20) (D57A2) (1:1000, Lot. 4199, Cell Signaling Technology), cleaved-IL-1β (D3A3Z) (1:1000, Lot. 83186, Cell Signaling Technology), Bcl-2 (D55G8) (1:1000, Lot. 4223, Cell Signaling Technology), Bax (D2E11) (1:1000, Lot. 5023, Cell Signaling Technology), Caspase3 (1:1000, Lot. 9662, Cell Signaling Technology), Cleaved-caspase3 (Asp175) (5A1E) (1:1000, Lot. 9664, Cell Signaling Technology), tyrosinase (TYR) (EPR10141) (1:1000, ab170905, Abcam), tyrosinase-related protein 1 (TYRP1) (EPR13063) (1:1000, Lot. ab178676, Abcam), micropthalmia-associated transcription factor (MITF) (1:1000, ab20663, Abcam), protease-activated receptor-2 (PAR-2) (1:1000, Lot. ab180953, Abcam), STAT3 (D3Z2G) (1:1000, Lot. 12640, Cell Signaling Technology), phospho-STAT3 (Tyr705) (D3A7) (1:1000, Lot. 9145, Cell Signaling Technology), Rab27a (1:1000, Lot. ab55667, Abcam) at 4°C overnight. After washing, the membranes were incubated with corresponding secondary antibodies (Goat Anti-Rabbit IgG Antibody, Peroxidase Conjugated, 1:5000, Lot. AP132P, Sigma-Aldrich, United States; Goat Anti-Mouse IgG Antibody, Peroxidase Conjugated, 1:5000, Lot. AP124P, Sigma-Aldrich, United States) for 1 h at room temperature. The bands were detected with an enhanced chemiluminescence reagent western blotting detection system (Cat. 871BRO7308, Bio-Rad, United States). The protein expression was quantified by using ImageJ 64 software and the band intensity was normalized to β-actin. Each western-blot assay was repeated at least for three times.

### Semi-Denaturing Detergent Agarose Gel Electrophoresis (SDD-AGE)

The SDD-AGE assay was conducted according to the established protocol (Hou et al., 2011). In short, crude cell lysis was resuspended in 1 × sample buffer (0.5 × TBE, 10% glycerol, 2% SDS, and 0.0025% bromophenol blue) and loaded onto a vertical 1.5% agarose gel (Bio-Rad, United States). After electrophoresis in the running buffer (1 × TBE and 0.1% SDS) for 35 min with a constant voltage of 100 V at 4°C, the proteins were transferred to Immobilon membrane (Millipore, Billerica, Mass) for immunoblotting and the following procedures are same as the Western-blot assay.

### Caspase1 Activity

Caspase1 activity was detected by Caspase1 Activity Assay Kit (Lot. C1102, Beyotime, China) according to the manufacturer’s instructions. In brief, cells were collected and lysed by lyase. Then, 40 μL detection buffer, 50 μL samples and 10 μL Ac-YVAD-pNAL-DOPA (2 mM) were added into 96-well plates and cultured for 2 h at 37°C. The caspase1 activity was quantified at 405 nm. The assay was performed in triplicate.

### Immunofluorescence

PIG1 cells were grown in single layer glass slides (Cat. 801002, NEST Biotechnology, China) at 5000 cells per dish. After washed with PBS and fixed with 4% paraformaldehyde, cells were incubated with Human IL-1R Fluorescein-conjugated antibody (5 μL, Lot. FAB269F, R&D Systems, United States) at 4°C overnight, protecting from light. The cells were subsequently washed twice with PBS and incubated with the nuclear dye 4′, 6-diamidino-2-phenylindole (DAPI) (1:1000, Lot. 62247, Thermo Fisher Scientific, United States) for 10 min at room temperature in dark. The fluorescence was detected by using FV-1000/ES laser confocal microscopy (Olympus, Tokyo, Japan).

### Annexin V-FITC/Propidium Iodide (PI) Apoptosis Assay

PIG1 cells were plated into 6-well plates at the density of 3 × 10^5^ cells each well and treated with the indicated treatments for 24 h. Cell apoptosis was detected by using the Annexin V-FITC/PI cell apoptosis kit (Lot. A005, 7sea biotec). PIG1 cells were collected and resuspended in 400 μL binding buffer, followed by the addition of 5 μL of Annexin V-FITC and 10 μL PI. After incubated for 15 min at room temperature, protecting from light, the apoptosis rates were measured by flow cytometry (Beckman Coulter, Miami, FL, United States) and analyzed with Expo32 software (Beckman Coulter, United States).

### Quantitative Real-Time Polymerase Chain Reaction (qRT-PCR)

Total RNA was extracted with TRIzol^TM^ Reagent (Lot. 15596018, Invitrogen, United States) and then reverse-transcribed to cDNA using a PrimeScript RT reagent kit (Lot. AK4301, TaKaRa, Japan). The qRT-PCR assay was performed using SYBR Premix Ex Taq II (Lot. AKA1008, TaKaRa, Japan) with the Real-time PCR Detection System (Cat. iQTM5, Bio-Rad, United States). Cycle threshold (CT) value was used to calculate the fold change by the 2^–ΔΔ*CT*^ method. The relative mRNA expression was normalized to β-actin. Primer sequences are listed as follows: Bcl-2 (forward 5′-TTGCCAGCCGGAACCTATG-3′ and reverse 5′-CGAAGGCGACCAGCAATGATA-3′), Bax (forward 5′-CCCGAGAGGTCTTTTTCCGAG-3′ and reverse 5′-CCAGCCCATGATGGTTCTGAT-3′), Caspase3 (forward 5′-AACCAGATCACAAAATTCTGCAAA-3′ and reverse 5′-TGGA GTCCAGTGAACTTTCTTCAG-3′), Cleaved-caspase3 (forward 5′-CCATAAAAGCACTGGAATGTCA-3′ and reverse 5′-CCG TTCGTTCCAAAAATTACTC-3′), TYRP1 (forward 5′-CACAA AACCACCTGGTTGAA-3′ and reverse 5′-CCAGCTTTGAAA AGTATGCC-3′), TYR (forward 5′-CCAGCCCATGATGGT TCTGAT-3′ and reverse 5′-GGCATTGTGCATGCTGCTT-3′), MITF (forward 5′-CGAAAGTTGCAACGRGAACAGC-3′ and reverse 5′-GAGCCTGCATTTCAAGTTCCTGT -3′), Rab27a (forward 5′-GCTTTGGGAGACTCTGGTGTA-3′ and reverse 5′-TCAATGCCCACTGTTGTGATAA-3′).

### Tyrosinase Activity

PIG1 cells were plated in 6-well plates at a density of 3.0 × 10^3^ cells per well, after culturing with corresponding HaCaT cell culture supernatant, the total protein was extracted and the concentration was measured with BCA Protein Assay kit (Pierce, Rockford, IL, United States). Then, 40 μL protein and 10 μL 3,4-Dihydroxy-L-phenylalanine (L-DOPA) (10 mM, V900425, Sigma-Aldrich) were added into 96-well plates and cultured for 30 min at 37°C. The tyrosinase activity was quantified at 500 nm. The assay was performed in triplicate.

### Statistical Analysis

Each statistical analysis was performed by GraphPad Prism 5 for Windows (United States) with two-tailed Student’s unpaired *t*-tests or one-way analysis of variance (ANOVA). These data are conformed to the normal distribution and the variance between the groups that are being statistically compared is similar. These data represent as mean ± SD for at least three independent experiments. ^∗^*P*< 0.05, ^∗∗^*P*< 0.01, ^∗∗∗^*P*< 0.001, NS = no significant, compared with the Ctrl group. ^#^*P*< 0.05, ^##^*P*< 0.01, ^###^*P*< 0.001, ns = no significant, compared with the H_2_O_2_ group.

## Results

### TR Mitigates the Secretion of IL-1β in Keratinocytes Under Oxidative Stress

**FIGURE 1:**
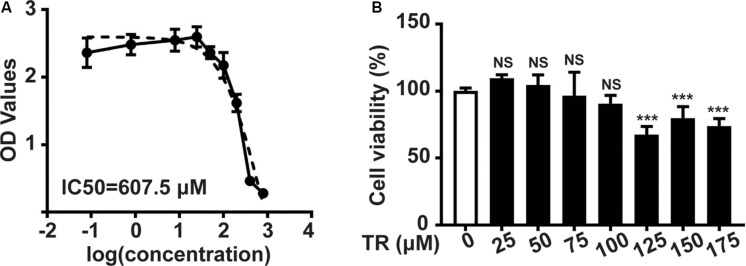
The safety concentration of TR in HaCaT cells is 100 μM. **(A)** The IC50 value of HaCaT cells treated by TR for 24 h, determined by CCK8 assay. **(B)** The cell viability of HaCaT cells treated by TR in the indicated concentration for 24 h, performed by CCK8 assay. Data represent the mean ± SD (*n* = 3). *P-*value was calculated by two-tailed Student’s *t*-test. ***P < 0.001, NS, not significant.

Initially, the CCK8 assay was performed to show that the IC50 (half maximal inhibitory concentration) of TR in HaCaT cells was 605.7 μM (Figure 1A). Sequentially, TR could inhibit the viability of HaCaT cells when the concentration rose to 125 μM (Figure 1B). Hence, 100 μM TR was selected to stimulate HaCaT cells in our following assay.

**FIGURE 2:**
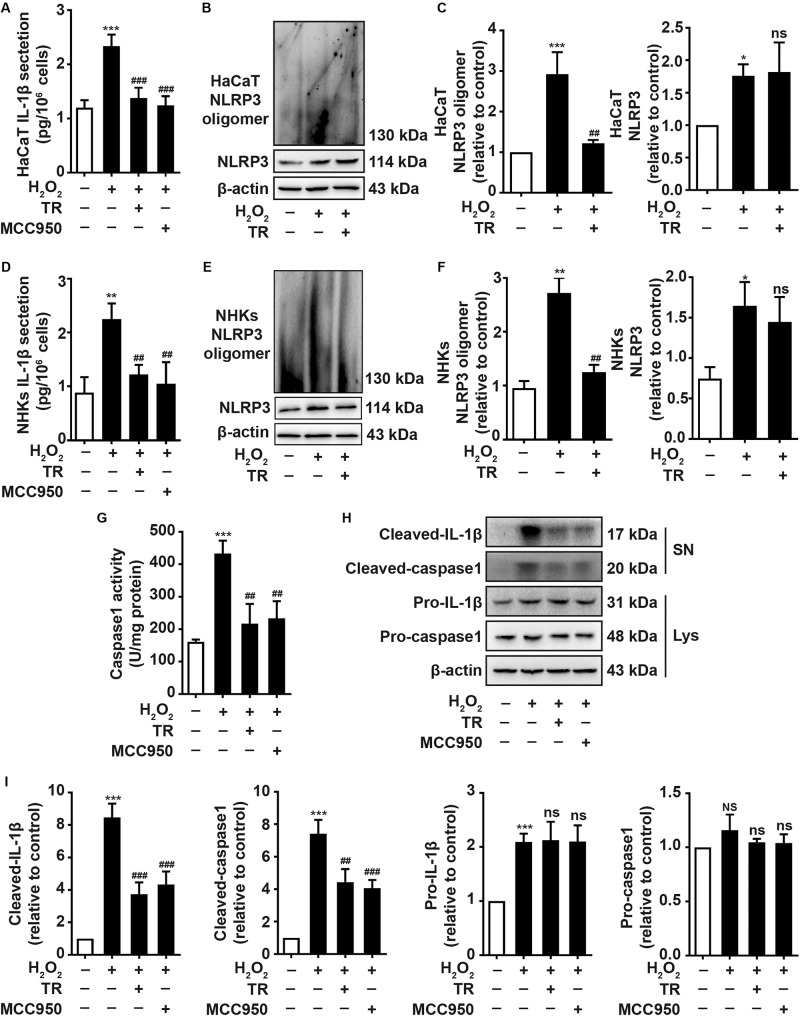
TR mitigates the secretion of IL-1β in keratinocytes under oxidative stress. **(A,D)** The secretion of IL-1β in HaCaT cells **(A)** and NHKs **(D)** treated by H_2_O_2_ or pretreatment with TR or MCC950 for 24 h prior to H_2_O_2_ disposition. **(B,C,E,F)** The alteration of assembly of NLRP3 treated with H_2_O_2_ for 24 h, or pretreated with TR prior to H_2_O_2_ disposition in HaCaT cells **(B,C)** and NHKs **(E,F)**, determined by SDD-AGE. **(G–I)** The detection of caspase-1 activity **(G)** and the expression of cleaved-IL-1β, cleaved-caspase-1, pro-IL-1β and pro-caspase-1 **(H,I)** in HaCaT cells when treated by H_2_O_2_ only or pretreated with TR or MCC950 prior to H_2_O_2_. Data represent the mean ± SD (*n* = 3). *P-*value was calculated by two-tailed Student’s *t*-test. *P < 0.05, **P < 0.01, ***P < 0.001, NS, not significant, compared with the Ctrl group. ^##^*P* < 0.01, ^###^*P* < 0.001, ns = not significant, compared with the H_2_O_2_ group.

To elucidate the impacts of TR on keratinocytes, ELISA results showed that TR could prominently compromise the increment of IL-1β elicited by H_2_O_2_ in HaCaT cells (Figure 2A) and NHKs (Figure 2D) and this effect was similar to the pretreatment of MCC950, a classical NLRP3 inhibitor (Figures 2A,D). To explore how TR lessened the production of IL-1β in response to H_2_O_2_ stimulation, our data manifested that TR treatment could effectively reverse the NLRP3 oligomerization which was apparently promoted by H_2_O_2_ in HaCaT cells (Figures 2B,C) and NHKs (Figures 2E,F), detected by the SDD-AGE assay. Consistently, TR lessened the caspase-1 activity promoted by H_2_O_2_, which was similar to the MCC950 (Figure 2G). What’s more, TR, resembling to the effect of MCC950, could decrease the expression of cleaved-IL-1β and cleaved-caspase1 which were elevated by H_2_O_2_, while the pro-IL-1β and pro-caspase-1 had no obvious alteration (Figures 2H,I). Taken together, these data indicated that TR could suppress the secretion of IL-1β by inhibiting the NLRP3 oligomerization in keratinocytes.

### TR Decreases the Melanocytes Apoptosis Through Lessening the Keratinocyte-Derived IL-1β Under Oxidative Stress

**FIGURE 3:**
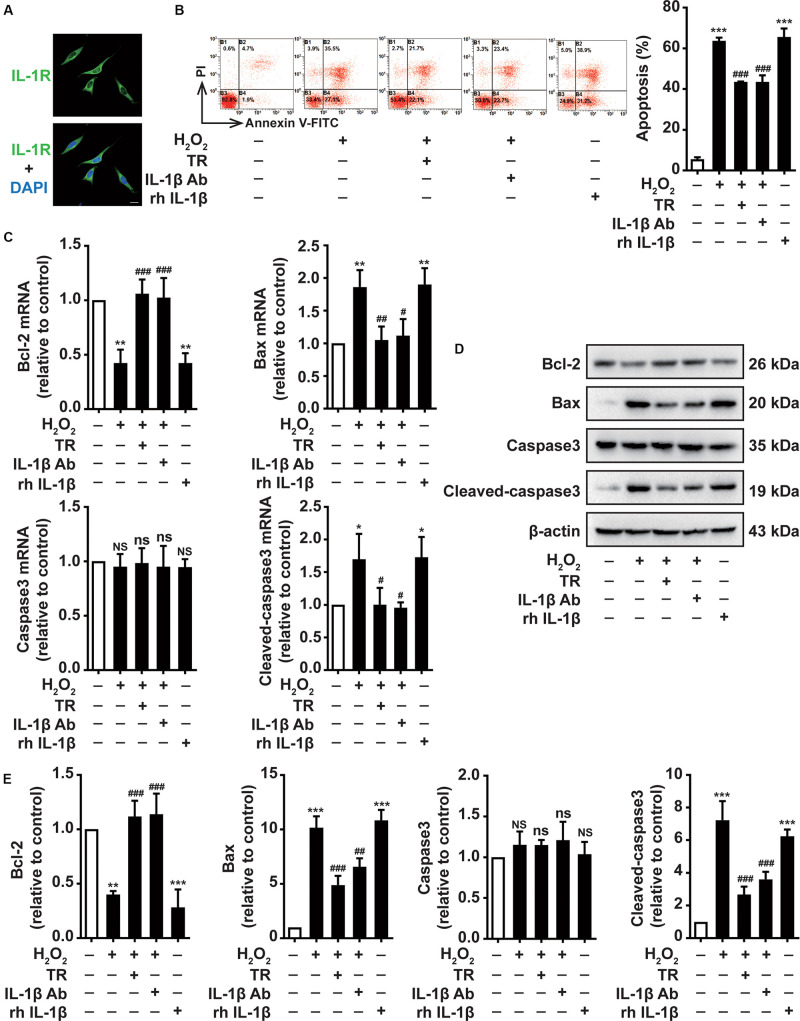
TR decreases the melanocytes apoptosis through lessening the keratinocyte-secreted IL-1β under oxidative stress. **(A)** The expression of IL-1R in the membrane of PIG1, Bar = 20 μm. **(B–E)** The apoptosis rate **(B)**, the mRNA **(C)**, and protein levels **(D,E)** of Bcl-2, Bax, caspase-3, and cleaved-caspase3 in PIG1 cells treated by rh IL-1β, or by the supernatant harvested from HaCaT cells pretreated by H_2_O_2_ only, or by TR prior to H_2_O_2_ stimulation, or by IL-1β neutralizing antibody after the H_2_O_2_ disposition. Data represent the mean ± SD (*n* = 3). *P-*value was calculated by two-tailed Student’s *t*-test. *P < 0.05, **P < 0.01, ***P < 0.001, NS, not significant, compared with the Ctrl group. ^#^*P* < 0.05, ^##^*P* < 0.01, ^###^*P* < 0.001, ns = not significant, compared with the H_2_O_2_ group.

Due to the close connection between keratinocytes and melanocytes, we next determined the effects of TR-treated keratinocytes supernatant on melanocytes. Above all, we suggested that the IL-1R was expressed in the membrane of PIG1 (Figure 3A). Then the supernatant harvested from the HaCaT cells was used to culture PIG1 cells. We showed that H_2_O_2_-stimulated HaCaT cells supernatant critically aggravated the PIG1 apoptosis, similar to the rh IL-1β impact on PIG1 (Figure 3B). Notably, the supernatant pretreated by TR prior to H_2_O_2_ disposition in keratinocytes significantly decreased the PIG1 apoptosis, which was similar to the addition of IL-1β neutralizing antibody in supernatant, detected by flow cytometry assay (Figure 3B). To probe the mechanisms underlying TR reversed the pro-apoptosis effect of HaCaT cells on PIG1, at both mRNA and protein level, we demonstrated that keratinocyte-secreted IL-1β could remarkably attenuate the expression of Bax and cleaved-caspase-3, while it could upregulate the expression of Bcl-2, which could be restored to normal levels by the pretreatment of TR in keratinocytes (Figures 3C–E). Altogether, these data deciphered that TR pretreatment in keratinocytes reversed the melanocytes apoptosis resulted from keratinocyte-deprivation IL-1β.

### TR Improves the Melanocytes Melanogenesis Through Abolishing the Keratinocyte-Derived IL-1β Under Oxidative Stress

**FIGURE 4:**
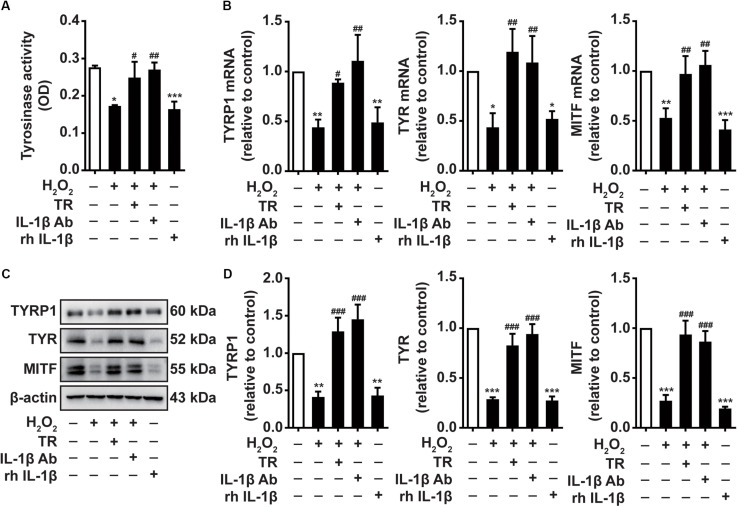
TR improves the melanocytes melanogenesis through abolishing the keratinocyte-secreted IL-1β under oxidative stress. **(A–D)** The tyrosinase activity **(A)** and the mRNA **(B)** and protein levels **(C,D)** of TYRP1, TYR, and MITF in PIG1 cells treated by rh IL-1β or the supernatant harvested from HaCaT cells pretreated by H_2_O_2_ only, or by TR prior to H_2_O_2_ stimulation, or by IL-1β neutralizing antibody after the H_2_O_2_ disposition. Data represent the mean ± SD (*n* = 3). *P-*value was calculated by two-tailed Student’s *t*-test. *P < 0.05, **P < 0.01, ***P < 0.001, NS, not significant, compared with the Ctrl group. ^#^*P* < 0.05, ^##^*P* < 0.01, ^###^*P* < 0.001, ns = not significant, compared with the H_2_O_2_ group.

Previous studies have attributed the great importance to tyrosinase activity in the melanocytes melanogenesis. Besides, the expression of TYRP1, TYR, and MITF is indispensable marker in the process of melanogenesis. We next investigate the effects of TR treatment in keratinocytes on the melanocytes melanogenesis, we exploited the supernatant harvested from HaCaT cells to culture PIG1 similarly. Intriguingly, we exhibited that HaCaT cells supernatant under H_2_O_2_ stimulation impaired the activity of tyrosinase, which was similar to the rh IL-1β disposition (Figure 4A). When TR pretreatment preceding H_2_O_2_ disposition in HaCaT cells or using IL-1β neutralizing antibody to impede IL-1β in supernatant, the impaired activity of tyrosinase was reversed to a normal status (Figure 4A). Furthermore, we displayed that H_2_O_2_-treated keratinocyte supernatant, resembling the rh IL-1β, could significantly abate the expression of TYRP1, TYR, and MITF at both mRNA and protein level in PIG1 cells (Figures 4B–D). Also, either TR pretreatment preceding H_2_O_2_ in HaCaT cells or using IL-1β neutralizing antibody to hinder IL-1β in supernatant restored the decreased expression of TYRP1, TYR and MITF at both mRNA and protein level (Figure 4B–D). These results underscored that TR improved the melanocytes melanogenesis via restraining the IL-1β production in keratinocytes.

### TR Has the Capacity to Optimize the Melanosome Translocation via Mediating the Keratinocyte Under Oxidative Stress

**FIGURE 5:**
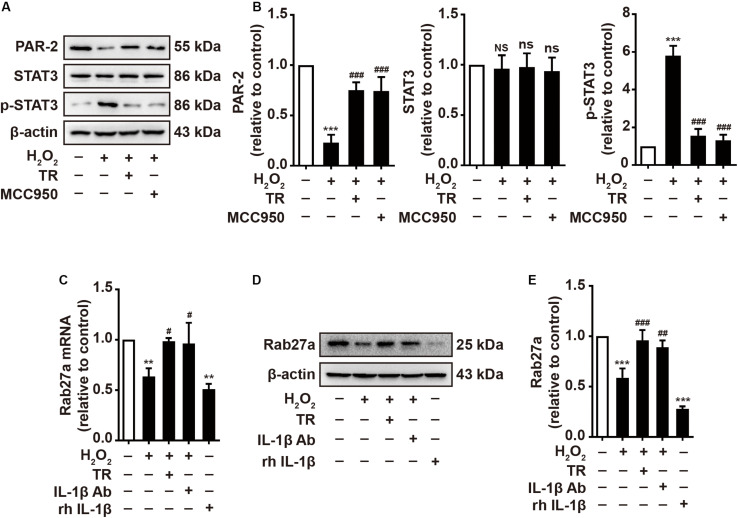
TR has the capacity to optimize the melanosome translocation under oxidative stress. **(A,B)** The expression of PAR-2, STAT3, p-STAT3 in HaCaT cells treated by H_2_O_2_ only, or by TR or MCC950 for 24 h prior to H_2_O_2_ stimulation. **(C–E)** The mRNA **(C)** and protein levels **(D,E)** of Rab27a in PIG1 cells stimulated by rh IL-1β, or the supernatant harvested from HaCaT cells pretreated by H_2_O_2_ only, or by TR prior to H_2_O_2_ stimulation, or by IL-1β neutralizing antibody after the H_2_O_2_ disposition. Data represent the mean ± SD (*n* = 3). *P-*value was calculated by two-tailed Student’s *t*-test. **P < 0.01, ***P < 0.001, NS, not significant, compared with the Ctrl group. ^#^*P* < 0.05, ^##^*P* < 0.01, ^###^*P* < 0.001, ns = not significant, compared with the H_2_O_2_ group.

Previous studies have pointed out that keratinocyte-expressed PAR-2 contributed importantly to the melanosome translocation (Seiberg et al., 2000). Hence, we manifested that H_2_O_2_ disposition reduced the expression of PAR-2 and activated STAT3 featuring the upregulation of phosphor-STAT3 which could mitigate the expression of PAR-2 (Figures 5A,B). Intriguingly, the TR treatment preceding H_2_O_2_ significantly restored the expression of PAR-2 and the activation of STAT3 in HaCaT cells, which is similar to the pretreatment of MCC950 (Figures 5A,B). Besides, it is reported that Rab27a, expressed in melanocytes, played a key role in the melanosome translocation (Jordens et al., 2006). Thus, we examined and illustrated that HaCaT-secreted supernatant with the H_2_O_2_ stimulation apparently decreased the Rab27a expression in melanocytes at both mRNA and protein level, which was similar to the effect of rh IL-1β (Figures 5C–E). Of note, when pretreated HaCaT cells with TR prior to H_2_O_2_ disposition or treated the supernatant with IL-1β neutralizing antibody, the supernatant could not attenuate the expression of Rab27a in melanocytes (Figures 5C–E). Taken together, these results verified that under oxidative stress, TR has the capacity to optimize the melanosome translocation defect by promoting the PAR-2 expression in keratinocytes, and potentiating the Rab27a in melanocytes via inhibiting the keratinocyte-derived IL-1β.

### TR Inhibits the Production of Inflammatory Cytokines in Keratinocytes Under Oxidative Stress

**FIGURE 6:**
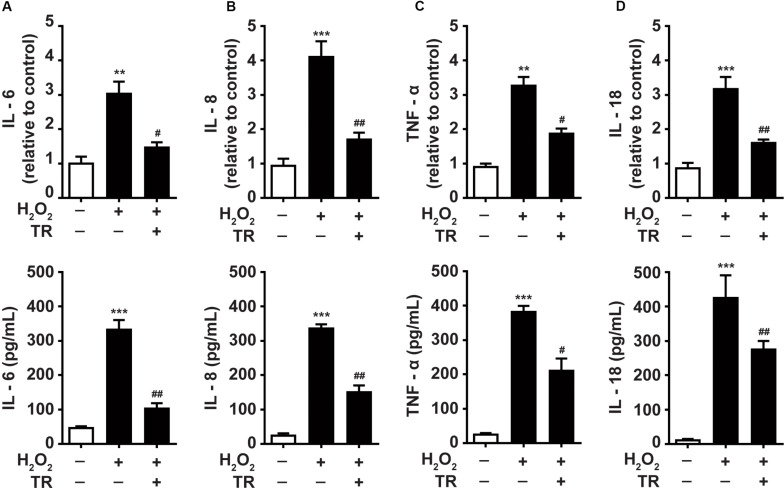
TR inhibits the production of inflammatory cytokines in keratinocytes under oxidative stress **(A–D).** The mRNA and protein of IL-6 **(A)**, IL-8 **(B)**, TNF-α **(C)**, IL-18 **(D)** in keratinocytes in response to H_2_O_2_ with or without the TR pretreatment. Data represent the mean ± SD (*n* = 3). *P-*value was calculated by two-tailed Student’s *t*-test. **P < 0.01, ***P < 0.001, compared with the Ctrl group. ^#^*P* < 0.05, ^##^*P* < 0.01, compared with the H_2_O_2_ group.

**FIGURE 7:**
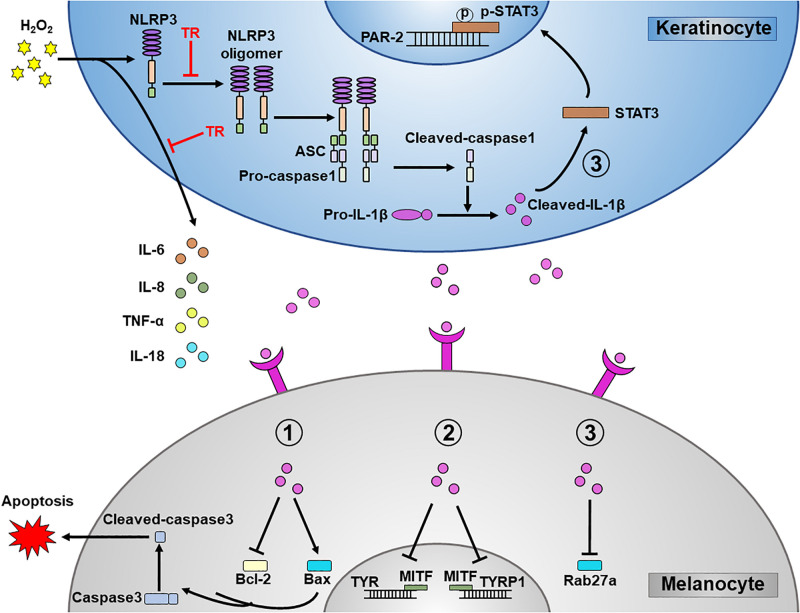
A schematic diagram. TR directly targets NLRP3 to protect melanocytes from keratinocyte-derived IL-1β which could aggravate the melanocyte apoptosis, interfere the melanogenesis and disturb the melanosome translocation. In addition, TR also impedes the secretion of pro-inflammatory cytokines such as IL-6, IL-8, TNF-α, and IL-18 in keratinocytes.

Previous studies underscored the upregulation of inflammatory cytokines in the pathogenesis of vitiligo (Dani et al., 2018; Gholijani et al., 2019). Hence, we detected and showed that at both mRNA and protein levels, the oxidative stress facilitated the expression of IL-6 (Figure 6A), IL-8 (Figure 6B), TNF-α (Figure 6C), and IL-18 (Figure 6D) in keratinocytes. Notably, the TR pretreatment could significantly palliate the increment of these cytokines elicited by oxidative stress in keratinocytes. These data suggested that TR could mitigate the severity of inflammation in the epidermis microenvironment under oxidative stress in vitiligo. Collectively, the present study unveiled that TR could ameliorate the detrimental impacts of keratinocyte-derived IL-1β on melanocytes under oxidative stress (Figure 7).

## Discussion

In the present study, we found that TR inhibited the activation of NLRP3 inflammasome by intervening its oligomerization in keratinocytes under the oxidative stress. More importantly, we verified that TR reversed the melanocyte apoptosis, improved the melanogenesis and have the capacity to optimize the melanosome translocation via mitigating IL-1β secretion from keratinocytes. In addition, TR could suppress the production of pro-inflammatory cytokines in keratinocytes under oxidative stress. The above supported TR as an effective therapeutic treatment for vitiligo.

The increasing evidences have supported the enormous role of oxidative stress in melanocyte loss in vitiligo. Oxidative stress can lead to various alterations of the antioxidant system, melanin synthesis–related proteins, and membrane lipids in melanocytes, leading to homeostasis disruption and survival compromise of melanocytes (Jian et al., 2014). Besides the oxidative stress impairs the homeostasis of melanocytes directly, it also exerts deleterious function on the microenvironment of epidermis to participate in the melanocyte destruction. As the predominant component in epidermis, keratinocyte is featuring as the close connection with melanocytes and the ability to produce a plethora of cytokines (Richmond et al., 2017). Previous studies have established that the dysfunction of keratinocytes under oxidative stress could generate excessive pathogenic chemokines such as CXCL9, CXCL10, CXCL11, and CXCL16 (Rashighi et al., 2014; Rashighi and Harris, 2015; Li et al., 2017; Richmond et al., 2017), which attracted autoreactive melanocyte-specific CD8^+^ T cells to the epidermis. All of these evidences indicated the colossal effects of keratinocytes on melanocytes in vitiligo pathogenesis under oxidative stress.

NLRP3 inflammasome is protein complexes which could sense different factors from either pathogens or hosts (Jiang et al., 2017). It is proved that NLRP3 inflammasome mediated several inflammatory diseases including type 2 diabetes (Masters et al., 2010), inflammatory bowel disease (Cocco et al., 2017) and neurodegenerative diseases (Heneka et al., 2013). In this study, we verified that under the oxidative stress, keratinocyte-derived IL-1β with the mediation of NLRP3 inflammasome could apparently aggravate the apoptosis of melanocytes and induce the defects of melanogenesis and melanosome translocation, which has been recognized as the important pathophysiology of vitiligo. In addition, our previous study illustrated that the expression of NLRP3 and downstream cytokine of IL-1β is upregulated in peripheral keratinocytes of vitiligo (Li et al., 2019). The elevated IL-1β not only enhance the chemotaxis of CXCL16-CXCR6 and CXCL10-CXCR3 in keratinocytes, but also could reinforce the function of melanocyte-specific CD8^+^ T cells (Li et al., 2019). To our knowledge, the existed off-table immunosuppress-targeting drugs is mainly focusing on the IFN-γ-JAK-chemokines signaling pathway, which exhibit the moderate effects in vitiligo therapy (Frisoli and Harris, 2017). Hence, targeting the NLRP3-IL-1β pathway may be another complementary and promising way in vitiligo treatment. Although the drugs targeting IL-1β have been used in some NLRP3-related diseases, the concerns regarding this treatment in vitiligo existed. First, IL-1β is not the only biological effect of NLRP3 inflammasome activation. The High-mobility group protein B1 (HMGB1), as a pro-inflammation factor in the downstream of NLRP3 inflammasome, has been proved to facilitate the secretion of IL-8 and CXCL16 in keratinocytes which contributed remarkably to the pathogenesis of vitiligo (Cui et al., 2019). Second, other inflammasomes or inflammasome-independent ways could also produce IL-1β (Levandowski et al., 2013), so inhibition of IL-1β function might have more immunosuppressive side-effects than inhibition of NLRP3 itself. Thus, the directly inhibiting NLRP3 inflammasome might be a better choice than targeting IL-1β for vitiligo treatment.

TR, as an analog of a tryptophan, could inhibit the homologous passive cutaneous anaphylaxis due to that it can palliate IgE-mediated histamine release from mast cells (Platten et al., 2005). Besides the canonic effect, TR has been applied in several inflammatory disease including bronchial asthma, atypical dermatitis, allergic conjunctivitis, and hypertrophic scars (Darakhshan and Pour, 2015) by directly binding to the NACHT domain of NLRP3 to suppress the of NLRP3 inflammasome (Huang et al., 2018). In line with this, we showed that the pretreatment of TR could significantly inhibit the assembly of NLRP3 inflammasome via impeding the NLRP3 oligomerization, and further decreased the apoptosis of melanocytes, improved the defects of melanogenesis and optimized the melanosome translocation via lessening the production of IL-1β in keratinocytes.

In terms of melanosome translocation, melanocytes are located in the basal layer and surrounded by ~36 keratinocytes in epidermis, which provides a physiological structure to the melanosome translocation (Van Den Bossche et al., 2006; Yamaguchi and Hearing, 2009). Previous results revealed that PAR-2 expressed in keratinocyte membrane is the receptor to catalyze the melanosome transfer, and the activation of STAT-3, featuring the upregulation of p-STAT3, is negatively regulator for PAR-2 (Seiberg et al., 2000). Besides, the expression of Rab27a in the membrane of melanocyte is also a promotor to facilitate the translocation of the melanosome (Jordens et al., 2006). Herein, our results exhibited that the NLRP3-mediated IL-1β could apparently restrain the expression of PAR-2 and Rab27a in keratinocytes and melanocytes respectively under the oxidative stress, which could be markedly eliminated by the pretreatment of TR. Moreover, TYR and TYRP1 could form multiple complexes on the internal surface of melanocytes (Cui et al., 2015), which synergistically increase the maturation of melanosomes. Notably, our data illustrated that the NLRP3-mediated IL-1β in keratinocytes could impair the melanosome synthesis by lowering the TYR activity and the expression of TYR and TYRP1, which could be impeded conspicuously by the pretreatment of TR in keratinocytes. Additionally, in this research, we also found that TR pretreatment could significantly palliate the severity of inflammation in epidermis under oxidative stress, including the decreased secretion of inflammatory cytokines such as IL-6, IL-8, TNF-α, and IL-18.

Importantly, compared to a few small-compounds targeting the NLRP3 inflammasome such as MCC950 (Dempsey et al., 2017), TR has been used in clinical practice due to its comparable effectiveness displayed in this assay, and the safety manifested by the fact that it is well tolerated by most patients at doses of up to 600 mg/day over months (Darakhshan and Pour, 2015). In consequence, we lifted TR as a promising therapeutic strategy for vitiligo.

In this study, we firstly verified that TR disturbed the NLRP3 oligomerization in keratinocytes under the oxidative stress and further inhibited the secretion of IL-1β. Sequentially, we validated that TR pretreatment in keratinocytes could decrease the melanocyte apoptosis and improve the melanogenesis and melanosome translocation via attenuating the secretion of IL-1β. Additionally, we found that TR pretreatment could significantly palliate the severity of inflammation in epidermis under oxidative stress. Given the *in vitro* model only in this research, further studies performed *in vivo* are still needed to confirm the role of TR in protecting melanocytes via regulating keratinocytes. In summary, our results underscored the promising therapeutic value of TR in vitiligo.

## Data Availability Statement

The original contributions presented in the study are included in the article/supplementary material, further inquiries can be directed to the corresponding authors.

## Author Contributions

TZ, PK, and CL conceived the project and designed the experiments. TZ, SL, XY, SG, and JC performed the experiments. TZ, YW, LL, and ZJ analyzed the data and wrote the manuscript. TG, PK, SG, and CL provided supervision, oversight, and leadership for the project. All authors contributed to review and editing of the manuscript.

## Conflict of Interest

The authors declare that the research was conducted in the absence of any commercial or financial relationships that could be construed as a potential conflict of interest.
